# The Daily Florida Union

**Published:** 1882-01-20

**Authors:** 


THE DAILY FLORIDA UNION is now among our exchanges. It is a large, neat and well managed paper, and speaks well for Jacksonville. It evinces unmistakable talent in its editorial columns. We are not surprised at this, since we have learned that Col. Jno. T. Graves, formerly of Atlanta, is now connected with its editorial department. Though a young man, he is a writer of fine ability and great promise. He is also an able and eloquent speaker.

